# Microstructure Development and Its Influence on the Properties of Styrene-Ethylene-Butylene-Styrene/Polystyrene Blends

**DOI:** 10.3390/polym10040400

**Published:** 2018-04-03

**Authors:** Ritima Banerjee, Suprakas Sinha Ray, Anup K. Ghosh

**Affiliations:** 1 Department of Materials Science & Engineering, Indian Institute of Technology Delhi, New Delhi 110016, India; ptz138489@polymers.iitd.ac.in; 2 DST-CSIR National Centre for Nanostructured Materials, Council for Scientific and Industrial Research, Pretoria 001, South Africa; 3 Department of Applied Chemistry, University of Johannesburg, Doornfontein 2028, Johannesburg 2018, South Africa

**Keywords:** polymer blends, morphology, fibrillar microstructure, rheology, mechanical properties

## Abstract

The present work is a novel attempt to understand the microstructure of styrene-ethylene-butylene-styrene (SEBS)/polystyrene (PS) blends not only through morphological studies, but also thermal, mechanical and rheological characterizations. SEBS/PS blends containing 10, 30 and 50 wt % PS were processed in a micro-compounder and characterized. Scanning electron microscopy (SEM) studies, with selective staining of the PS phase, revealed the presence of PS as nanometer-sized domains, as well as phase-separated micrometer-sized aggregates. Blends with 30 and 50 wt % PS exhibited a fibrillar microstructure, obeying Hirsch’s model of short fiber composites. A remarkable increase in glass transition temperature indicated a strong interaction of the fibrils with SEBS. All blends showed two modes of relaxation corresponding to the two phases. A single mode of relaxation of the PS phase has been attributed to combined effects of the partial miscibility of the added PS, along with the interaction of the fibrils with SEBS. The long relaxation time of the elastomeric phase indicated the tendency of these materials to undergo time-dependent shrinkage in secondary processing operations. An increase in PS content resulted in the lowering of the shear viscosity and energy requirement for mixing, indicating the ease of processing.

## 1. Introduction

Thermoplastic elastomers (TPEs), which have the elastic behavior of thermoset rubbers and the re-usability of thermoplastics, are an important class of materials.

Among the TPEs, styrene-ethylene-butylene-styrene (SEBS) has gained significant commercial importance because of its superior thermal and weathering resistance, attributed to the saturation of its elastomeric mid-block [1]. However, in spite of having advantages over thermoset rubbers, SEBS seldom finds application as a neat polymer [2]. This is because its mechanical properties are lower than those of thermoset rubbers. 

The mechanical properties of SEBS may be enhanced by blending with homopolymers [3]. When the added homopolymer is polystyrene (PS), improvement of the properties may be achieved without the use of an external compatibilizer. However, the extent to which this objective is met will depend on the microstructure of the blend. SEBS, like any other TPEs, has a complex microstructure, comprised of two phases, the thermoplastic phase and the rubber phase, both phases having widely different rheological properties. This complexity is further enhanced in blends, in which there is an additional component. Further, if the additional component is PS, which is one of the components of the block copolymer itself, the miscibility of the PS will also have a crucial role in determining the properties of the blend [3,4]. However, these complex-structured blends score high in terms of the stability of the microstructure. Veenstra et al. [5] have shown that the coarsening of these materials is severely slowed down and even arrested by physical cross-links, a characteristic feature of TPEs.

The properties of all immiscible blends are governed by the shape, size and distribution of the dispersed phase, as well as interfacial interactions. A blend containing well-dispersed spheres will exhibit significant improvement of impact properties as compared to matrix polymer; a blend with a fibrillar morphology will exhibit superior unidirectional tensile properties; and one with sheet-like inclusions will possess superior barrier properties [5,6,7]. In a co-continuous morphology, both components can fully contribute to the properties of the blend [5]. Since the objective of preparing SEBS/PS blends is enhancement of the tensile properties, the development of blends with a well-dispersed fibrillar morphology will help in accomplishing this goal. Understanding the properties of these blends in correlation with the microstructure will provide the knowledge necessary for tailoring properties to meet requirements.

Dynamic rheology is an important tool in the evaluation of the microstructure of multi-phase polymeric materials. Maani et al. [8] and Banerjee et al. [9] reported that for blends of a given composition, a finer morphology of the dispersed phase results in higher values of complex viscosity and storage modulus in melt state rheological studies. Ray et al. [10,11,12] have attributed the absence of cross-over frequency to pseudo-solid like behavior caused by prevented relaxation. Yu et al. [13] have correlated relaxation processes to changes in the microstructure of the polymer. Researchers [14,15,16,17] have also used the relaxation spectrum to study interfacial interactions in polymer blends.

Shear rheology, though less important in the evaluation of microstructure, has also been used to understand flow behavior in terms of blend morphology. Afshari et al. [18], in their studies on polypropylene/nylon 6 blends, correlated the deviation of viscosity from the value obtained using the additive rule of mixtures to the change in microstructure brought about by compatibilization. Other research groups [19,20,21] have also correlated the interactions between blend components to shear viscosity.

In addition to providing insight into the microstructure of polymeric materials, rheology also provides information related to the ease of processing, energy requirements and shrinkage after secondary processing operations. A lower shear viscosity has been associated with greater ease of flow [22] and higher molecular mobility [23]. A higher extent of shear thinning behavior leads to lower shear viscosity during mold filling, which in turn results in lowering of the energy requirement for molding [24,25]. Manchado et al. [26] correlated the steady state torque values achieved during mixing to the viscosity of the melt, showing that a lowering of viscosity led to lower values of torque and lower consumption of energy during mixing. Relaxation time is an indicator of residual frozen-in stresses in the product [27]. A long relaxation time would result in higher frozen-in stresses, which would affect product performance through time-dependent shrinkage. 

This work investigates the effect of blending SEBS with PS, which is one of the components of the block copolymer itself. It describes the development of SEBS/PS blends of superior mechanical properties by tailoring the microstructure through the change in the PS content of the blends. These blends have potential for application in breathable packaging applications, where SEBS would allow permeation of gases such as oxygen and carbon dioxide while providing protection against moisture [28,29], whereas PS would serve to increase the mechanical properties and also lower the cost. Blending with PS would enhance the stiffness (modulus) and tensile strength of SEBS, thus increasing the ability to resist deformation and withstand load during transportation and handling. 

The novelty of this work lies in its attempt to gain an understanding of the microstructure of SEBS/PS blends and the associated interactions of the phases not only through morphological studies, but also through thermal, mechanical and rheological characterizations. The work reports the development of fibrillar morphology, in which the added PS has a strong interaction with SEBS. Mechanical modelling has been used to support the findings of morphological analysis. Rheological studies and torque measurements have also been used to estimate the ease of processing and energy requirements. This paper is expected to provide guidelines in the production of TPE-based materials of superior mechanical properties.

## 2. Materials and Methods

### 2.1. Raw Materials

SEBS (Kraton G1643 M, reported to have 20 wt % styrene blocks) of Kraton Polymers India (Mumbai, India) and PS (SC202EF) of Supreme Petrochemicals India (Delhi, India) were used in this study. SEBS and PS were found to have melt flow index (MFI) values of 17.95 g/10 min (230 °C, 2.16 kg) and 5.17 g/10 min (200 °C, 5 kg), respectively.

### 2.2. Blend Sample Preparation and Torque Measurements

SEBS/PS blends having various compositions (Table 1) were processed using a Thermo Haake MiniLab conical co-rotating twin-screw micro-compounder (Thermo Fisher Scientific, Waltham, MA, USA). Screw diameters of the micro-compounder were 14 and 5 mm in the feed zone and die zone, respectively, and the channel depth was constant (0.25 mm). Processing was carried out at an 80-rpm screw speed, 200 °C barrel temperature and 3-min residence time (after material filling).

The steady state torque value attained during compounding was recorded for all compositions. Torque measurements were carried out for compounding 5 g of every composition. The torque value without any sample (*T*_2_) at the same screw speed and temperature was noted for calibration purposes. The effect of compositional variation on the steady state torque value was obtained from the values of *T*_s_, calculated using Equation (1):

(1) $$T_{s}=T_{1}-T_{2}$$

where *T*_1_ is the steady state torque reading before calibration.

The extruded strands were directly fed into the cylinder of a micro-injection molding machine HAAKE MiniJet II (Thermo Fisher Scientific, Waltham, MA, USA) and molded into tensile specimens of gauge dimensions 7.62 × 3.2 × 3.2 mm^3^, which were used for the evaluation of mechanical properties. Injection molding was performed using a cylinder temperature of 210 °C, mold temperature of 30 °C and injection pressure of 510 bar. Neat SEBS was processed in the micro-compounder and molded using the same parameters so as to achieve the same processing history.

### 2.3. Morphological Studies

**Figure 1:**
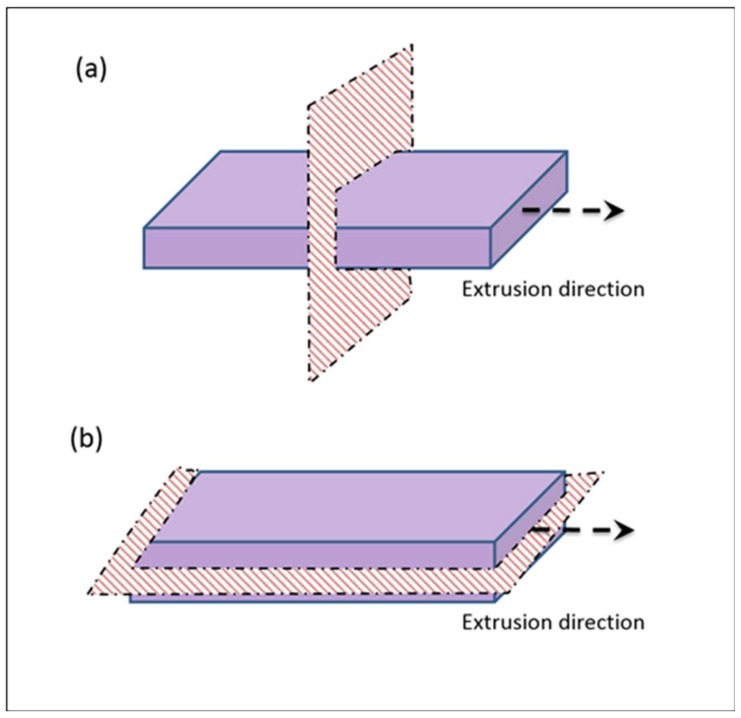
Cryo-fracturing of specimens (**a**) perpendicular to the direction of extrusion and (**b**) along the direction of extrusion.

Extruded strands of SEBS and the blends were cryo-fractured inside liquid nitrogen, perpendicular to the direction of extrusion (Figure 1a), as well as along the direction of extrusion (Figure 1b). Selective staining of the PS phase was carried out by exposing the cryo-fractured samples for 1 h to vapors of freshly-prepared 5% RuO_4_ solution. The stained samples were carbon coated and subjected to SEM studies using an FEI Quanta 200 instrument (FEI, Hillsboro, OR, USA), using back-scattered electron imaging mode, so as to get a contrast based on difference in chemical compositions.

### 2.4. Tensile Test

Tensile tests on the blends and neat SEBS were performed with a ZWICK universal tester (Zwick Roell Group, Ulm, Germany), Model 2010. A cross-head speed of 500 mm/min (a typical value used for testing neat SEBS [30,31]) was used for testing all samples so as to evaluate the effect of blending different proportions of PS with neat SEBS. Five specimens of each sample were tested, and the average values of moduli at 20% strain (*E*_20_), tensile strength (UTS) and elongation at break (EAB) have been reported. Toughness was calculated from the area under the stress-strain curves. Blending with a rigid thermoplastic like PS is expected to significantly enhance the modulus at lower strains. Even engineering applications of elastomeric materials often involve strains not more than 20% [32]. Furthermore, not very high strains are expected during transportation and handling of packaging materials, a possible application of these materials. Taking into account all these factors, a strain of 20% was chosen for the determination of the modulus. At lower strains, the modulus will be superior to *E*_20_ and the product performance will be better.

### 2.5. Thermal Characterization

The glass transition temperatures (*T*_g_) and temperatures of the extrapolated onset of thermal degradation (*T*_onset_) of the SEBS, PS and the blends were determined from differential scanning calorimetry (DSC) and thermal gravimetric analysis (TGA), respectively, so as to confirm the suitability of the temperatures used for primary and secondary processing operations. 

DSC studies were carried out using a DSC Q 200 TA instrument (New Castle, DE 19720, USA). The samples were heated from ambient temperature to 150 °C at 10 °C/min. Temperature was maintained at 150 °C for 5 min so as to eliminate thermal history. This was followed by cooling to −80 °C at 10 °C/min and reheating in continuity to 110 °C at 10 °C/min, so as to determine the *T*_g_. The *T*_g_ values obtained from DSC studies were compared with the corresponding values obtained from loss modulus peaks in the Dynamic Mechanical Analyzer (DMA) (Q200 Dynamic Mechanical Analyzer of TA Instruments, New Castle, DE 19720, USA). temperature sweep carried out in the range −60–120 °C at a frequency of 1 Hz, using a strain of 0.057% and a temperature ramp of 5 °C/min. The *T*_g_ values were also used to gain an understanding of the morphology of the blends.

TGA studies were carried out using a Perkin Elmer Pyris 6 TGA instrument (PerkinElmer, Inc., Waltham, MA, USA). The samples were heated from room temperature to 800 °C at a rate of 20 °C/min under nitrogen atmosphere. The temperatures corresponding to extrapolated onset of degradation (*T*_onset_), 25% weight loss (*T*_25_) and 50% weight loss (*T*_50_) were used for comparison of the thermal stability of the materials.

### 2.6. Melt State Dynamic Rheological Studies

Studies on the melt-state dynamic rheological properties of SEBS, PS and the blends were performed using a parallel plate rotational rheometer (Anton Paar MCR 302, P-ETD 400) (Anton Paar GmbH, Graz, Austria) having a 25-mm plate diameter, using 1 mm-thick discs, prepared by compression molding at a temperature of 210 °C and pressure of 15,000 lbf for 5 min. The frequency sweep was performed in the range 0.01–100 rad·s^−1^, at 200 °C, using a strain of 0.5%, after determining the linear viscoelastic (LVE) region at the same temperature. LVE was determined by carrying out the amplitude sweep at a frequency of 10 rad/s.

### 2.7. Shear Rheology

**Figure 2:**
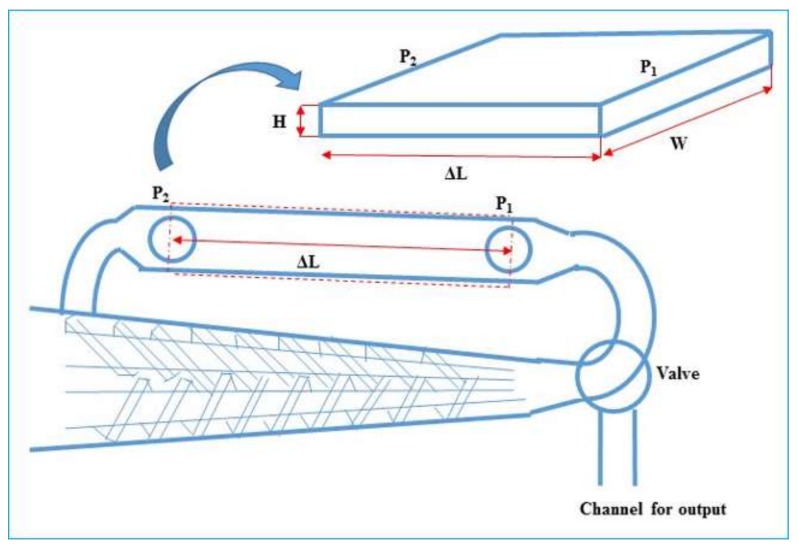
Schematic diagram of the portion of micro-compounder having a slit capillary back-flow channel, which can be used for rheological studies.

Shear rheological studies on SEBS, PS and the blends were performed in the same micro-compounder that was used for the preparation of the blends. The MiniLab has a backflow channel with the design of a slit capillary (Figure 2). 

Pressure is measured at the capillary entrance and exit. The shear stress (τ) and the apparent shear rate ($\dot{\gamma }$_app_) in the capillary and in the resultant apparent viscosity (η_app_) are given by Equations (2)–(4):

(2) $$\tau =\frac{h}{2\Delta L}\Delta P$$

(3) $${\dot{\gamma }}_{\mathrm{app}}=\left(\frac{6}{wh^{2}}\right)Q_{v}$$

(4) $$\eta_{\mathrm{app}}=\frac{\tau }{{\dot{\gamma }}_{\mathrm{app}}}$$

where *h* is the height of the flow channel (1.5 mm), *w* is the capillary width (10 mm), Δ*L* is the length of the capillary between the two pressure sensors (64 mm) and Δ*P* is the pressure drop along the capillary length Δ*L*. *Q*_v_ is the volumetric flow rate through the flat die and can be calculated using Equation (5), which has been proposed by the micro-compounder manufacturer:

(5) $$Q_{v}=C\cdot \Omega$$

where Ω is the screw rotation speed and *C* is a calibration constant whose default value as set in the software is 8 × 10^−7^ m^3^. This value has been calculated for a polyolefin material and is not valid for all materials, as shown by Yousfi et al. [33]. However, this *C* value has been used by various research groups [34,35,36] to calculate relative viscosities of different formulations for comparison purposes, as proposed by the manufacturer. The same approach has been employed in the present studies. 

During rheometry measurements, the micro-compounder was set in “cycle” mode, so as to direct all material into the slit channel. Temperature was set at 200 °C. Material was fed manually into the inlet of the instrument. Screw speed was initially set at 10 rpm and increased up to 350 rpm, in 10 stages. Corresponding to every screw speed, the values of apparent shear rate and apparent viscosity, as obtained by measurement of equilibrium pressure and using HAAKE Polylab (which employs Equations (2)–(5)), were recorded.

## 3. Results and Discussion

### 3.1. Morphological Studies

**Figure 3:**
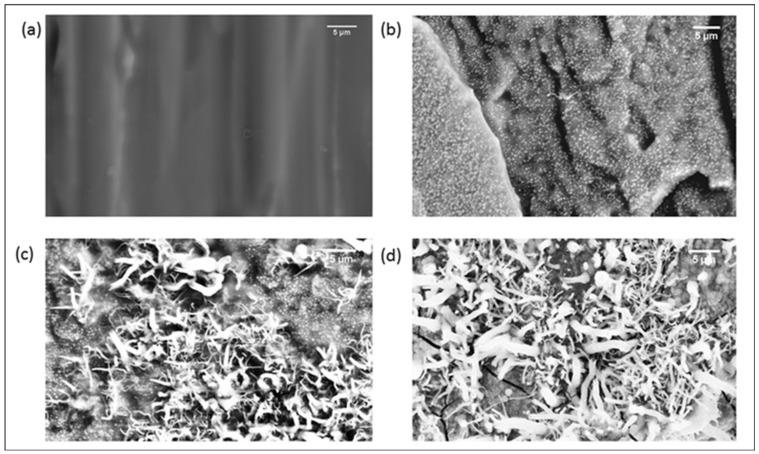
SEM images at 5 k magnification of (**a**) SEBS (**b**) SEBS-10PS (**c**) SEBS-30PS and (**d**) SEBS-50PS specimens cryo-fractured perpendicular to the direction of extrusion.

**Figure 4:**
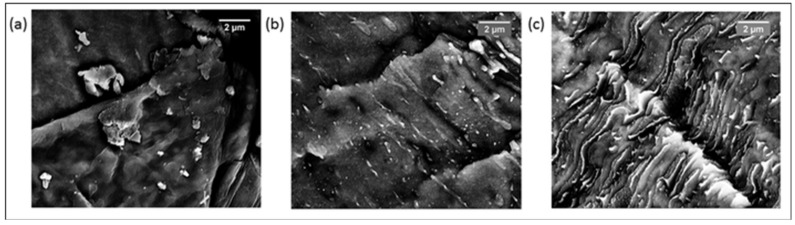
SEM images at 20 k magnification of (**a**) SEBS-10PS (**b**) SEBS-30PS and (**c**) SEBS-50PS specimens cryo-fractured along the direction of extrusion.

Figure 3 shows the SEM images of specimens, which were cryo-fractured perpendicular to the direction of extrusion. It has been established through TEM and AFM studies [1,2,3,31] that in grades of SEBS with PS as the minor component, above the *T*_g_ of PS, the PS forms nanometer-sized domains embedded in the elastomeric phase. However, in the SEM micrograph of SEBS (Figure 3a), PS domains are not visible at the magnification used for the studies. However, at the same magnification, whitish nanometer-sized particles are observed in the case of SEBS-10PS blend (Figure 3b), indicating an increase in domain size. A few micrometer-sized particles are also observed, which are obvious in specimens cryo-fractured along the direction of extrusion (Figure 4a). Thus, the added PS was partially miscible in the PS domains of SEBS, leading to an increase in the domain size. The remaining portion became phase separated, resulting in the formation of micrometer-sized aggregates. Such partial miscibility of PS in SEBS/PS blends has also been reported by other research groups [3,37]. An increase in PS content led to an increase in the number of phase separated aggregates (Figure 3c,d). In SEBS-30PS (Figure 3c) and SEBS-50PS (Figure 3d), the phase separated PS is in the form of fibrils. These fibrils can also be observed in the specimens that were cryo-fractured along the direction of extrusion (Figure 4b,c).

In SEBS-50PS (Figure 4c), some of the fibrils are found to be extending continuously along the length of the specimen and are interconnected with each other, indicating a co-continuous morphology. Such a fibrillar morphology with co-continuity is expected to have an enhanced synergistic combination of the properties of SEBS and the superior stiffness of PS, as compared to blends of a droplet/matrix morphology [5], making it more suitable for rigid applications. The significant enhancement of mechanical properties can be observed in the subsequent section of the paper.

### 3.2. Mechanical Properties

**Figure 5:**
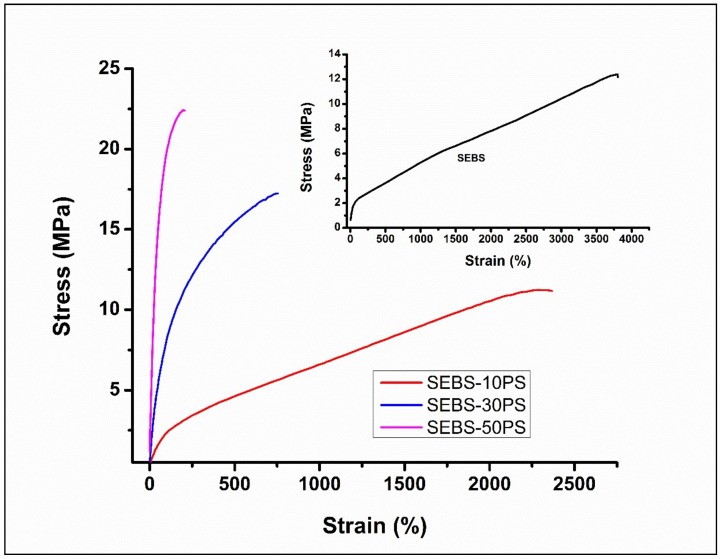
Representative tensile curves of SEBS and the blends.

Figure 5 shows the representative tensile curves of the SEBS blends. It can be observed that in all compositions, the slope of the curves changes, indicating a strain-dependent modulus, as in the case of typical elastomeric materials.

The tensile properties are given in Table 1. As compared to SEBS, SEBS-10PS has a slightly lower value of *E*_20_ and comparable UTS. This is possibly due to the larger domain size [37]. The block copolymer type of TPEs behave like cured rubbers at lower temperatures because of microphase separation of the immiscible blocks. The microphases possess dimensions of the order of the dimensions of polymer molecules, which results in a highly organized domain morphology [1,2]. An increase in domain size adversely affects the inherent morphology of SEBS, resulting in deterioration of the properties [38] and, hence, a lower *E*_20_ value. A further increase in PS content results in a remarkable increase in *E*_20_ (the *E*_20_ of SEBS-50Ps is almost 11-times higher than that of SEBS) along with enhancement of UTS. Such a large increase may be attributed to the over-riding effect of the increased number of phase separated PS aggregates. In SEBS-30PS and SEBS-50PS, the phase separated PS is in the form of fibrils aligned in the flow direction (Figure 4b,c). These fibrils are more effective than the small number of randomly-distributed and irregularly-shaped aggregates in SEBS-10PS (Figure 4a) in enhancing mechanical properties. Furthermore, the aligned fibrils exhibit a strong interaction with SEBS, as suggested by *T*_g_ and relaxation time measurements (discussed subsequently), further enhancing the mechanical properties. The co-continuity observed in SEBS-50PS is another factor contributing to mechanical reinforcement.

The experimental values of the modulus were compared with the predictions of the series model (Equation (6)), parallel model (Equation (7)), Takayanagi Models I and II (Equations (8) and (9), respectively), the Davies model (Equation (10)) and Hirsch’s model (Equation (11)).

(6) $$E={\left(\frac{\varphi_{\mathrm{PS}}}{E_{\mathrm{PS}}}+\frac{\varphi_{\mathrm{SEBS}}}{E_{\mathrm{SEBS}}}\right)}^{-1}$$

(7) $$E=\varphi_{\mathrm{PS}}E_{\mathrm{PS}}+\varphi_{\mathrm{SEBS}}E_{\mathrm{SEBS}}$$

(8) $$E=\lambda^{2}{\left(\frac{\lambda }{E_{\mathrm{PS}}}+\frac{1-\lambda }{E_{SEBS}}\right)}^{-1}+\left(1-\lambda^{2}\right)E_{SEBS}$$

(9) $$E={\left(\frac{\lambda }{\lambda^{2}E_{\mathrm{PS}}+\left(1-\lambda^{2}\right)E_{\mathrm{SEBS}}}+\frac{1-\lambda }{E_{\mathrm{SEBS}}}\right)}^{-1}$$

(10) E15=φPSEPS15+φSEBSESEBS15

(11) $$E=x\left(\varphi_{\mathrm{PS}}E_{\mathrm{PS}}+\varphi_{\mathrm{SEBS}}E_{\mathrm{SEBS}}\right)+\left(1-x\right)\;{\left(\frac{\varphi_{\mathrm{PS}}}{E_{\mathrm{PS}}}+\frac{\varphi_{\mathrm{SEBS}}}{E_{\mathrm{SEBS}}}\right)}^{-1}$$

where *E*, *E*_PS_, *E*_SEBS_ are the tensile modulus values of the blend, PS (*E*_PS_ was found to be 732.61 MPa) and SEBS, respectively, φ_PS_ and φ_SEBS_ are the volume fractions of PS and SEBS, respectively, λ is the length fraction of the discrete phase (PS) and is given by Equation (12), while *x* in Hirsch’s model represents the contribution of the parallel model and has been explained subsequently.

(12) $$\lambda =\varphi_{\mathrm{PS}}{}^{1/3}$$

The Takayanagi models [39,40,41] are useful in predicting tensile modulus of blends with a droplet/matrix morphology. The Davies model [42] of dual phase co-continuity is useful in predicting the modulus of blends with colony-type co-continuous morphology [28].

Hirsch’s model is widely used for predicting the tensile modulus of short fiber composites. It is a combination of the series and the parallel models. The parameter *x* indicates the stress transfer between the fibers and the matrix. The value of *x* depends on the length and orientation of the fibers, as well as the stress amplification effect at the ends of the fibers [43,44].

**Figure 6:**
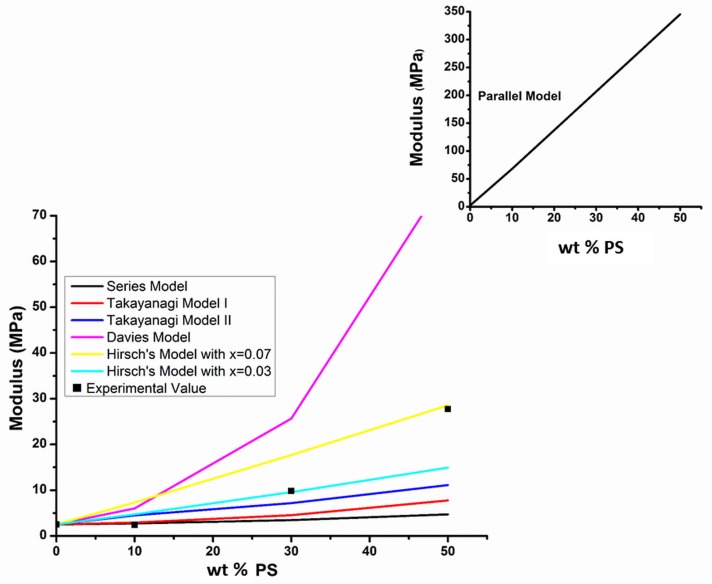
Comparison of the experimentally-determined tensile modulus values of the blends with the predictions of the models.

Figure 6 shows the comparison of the experimentally-determined modulus values of the blends with the predictions of the models. All experimental values were found to lie between the values predicted by the series and the parallel models, which represent the lower and upper boundaries of predicted values. An increase in PS content of the blends resulted in a greater deviation from the series model. SEBS-30PS and SEBS-50PS were found to possess modulus values higher than the predictions of both Takayanagi models, indicating a morphology different from the droplet/matrix morphology, thus indirectly supporting the observations of SEM studies regarding the development of the fibrillar morphology. The modulus value of SEBS-50PS was lower than the prediction of the Davies model, despite the fact that the SEM micrograph (Figure 4c) of SEBS-50PS showed inter-connected fibrils indicative of co-continuity. This anomaly may be attributed to the fact that the morphology of SEBS-50PS as observed in the SEM micrographs (Figure 3d and Figure 4c) was not colony-like co-continuous, but fibrillar. In fact, both SEBS-30PS and SEBS-50PS were found to follow Hirsch’s model, though for different values of *x*. SEBS-30PS and SEBS-50PS followed Hirsch’s model at *x* values of 0.03 and 0.07, respectively. Some of the factors responsible for the difference in *x* values may possibly be the difference in fibril lengths of SEBS-30PS and SEBS-50PS, as well as the inter-connections between the fibrils of SEBS-50PS (Figure 4b,c).

The tensile curves have other interesting features. It can be observed that in all compositions, the slope of the curves changes with strain, indicating a strain-dependent modulus, as in the case of typical elastomeric materials. An increase in PS content leads to a change in the shape of the curve from that of a typical elastomeric material to that of a thermoplastic with a significant lowering of elongation at break (EAB of SEBS is almost 19-times higher than that of SEBS-50PS) and an increase in tensile strength (UTS of SEBS-50PS is about twice that of SEBS), in addition to the increase in stiffness. The increase in tensile strength indicates an increase in the ability to sustain load without breaking. The fact that the tensile properties (shape of curve, values of UTS, EAB and *E*_20_) of SEBS-30PS and SEBS-50PS are significantly different from those of SEBS and SEBS-10PS, which behave like typical elastomeric materials, can be attributed to the development of a fibrillar morphology in which the phase separated PS can significantly influence the tensile properties.

The combined effect of the increase in modulus and tensile strength along with the reduction in elongation at break was a lowering of the toughness. 

The value of EAB of SEBS-50PS is around 200%. Interestingly, despite being much lower than that of neat SEBS, it is still reasonably high and comparable to that of some cured synthetic rubbers [45], thus indicating a significant extent of ductility despite a remarkable increase in stiffness.

### 3.3. Thermal Characterization 

**Figure 7:**
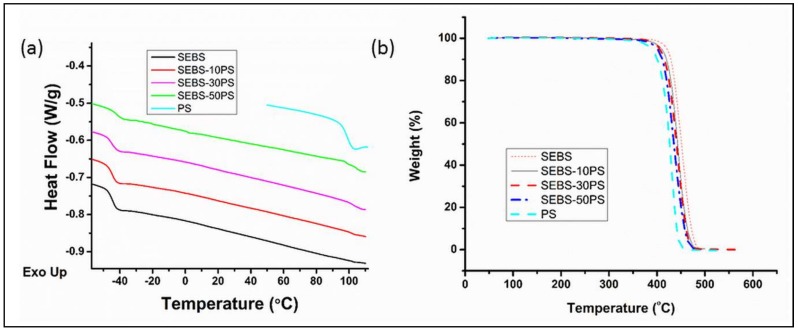
(**a**) Differential scanning calorimetry curves and (**b**) TGA curves.

Figure 7a shows the DSC plots of SEBS, PS and the blends. The *T*_g_ values of the ethylene-butylene (EB) and PS phases obtained from the step change in DSC plots and the peaks in loss modulus plots [38] are given in Table 2.

Although the SEM micrographs of the blends revealed an increase in domain size, as well as the presence of phase separated aggregates, indicating partial miscibility of the added PS with the PS domains of SEBS, all blends were found to possess a single *T*_g_ of the PS phase in the DSC and DMA studies. This is contrary to the expected presence of multiple *T*_g_s in a phase separated (immiscible/partially miscible) polymer blend, in which each component exhibits its own glass transition temperature. This anomaly can be explained on the basis of the fact that when *T*_g_s of the individual components varies by less than 20 K, a single *T*_g_ can appear even in phase-separated systems [46,47], and in the present studies, the difference between *T*_g_ of the PS phase of the copolymer (*T*_g_-cPS) and the *T*_g_ of the homopolymer (*T*_g_-hPS) was less than 10 K.

From the loss modulus plots, the *T*_g_ of the PS phase of SEBS-10PS was found to lie between *T*_g_-cPS and *T*_g_-hPS, as in case of miscible blends. This can be explained on the basis of the predominance of the effect of increased domain size, as also observed in the *E*_20_ value, as well as earlier studies [37,38].

However, in SEBS-30PS and SEBS-50PS, in which the effect of phase separated PS fibrils was expected to predominate [37,38], the *T*_g_ of the PS phase, as determined from the loss modulus peaks, was higher than both *T*_g_-cPS and *T*_g_-hPS. *T*_g_ of the PS phase of SEBS-30PS was found to be 10 °C higher than *T*_g_-hPS and 23 °C higher than *T*_g_-cPS. Such a large positive shift of *T*_g_ is unusual in blends and is indicative of a strong interaction of the components [46,48], which results in hindered segmental mobility of PS in the blends, as compared to the neat polymer. Strong interaction of the phase separated PS with SEBS has also been suggested by the weighted relaxation spectrum results, discussed subsequently. Despite having a higher weight fraction of PS, SEBS-50PS exhibits 4 °C lower *T*_g_ (determined from loss modulus peak) of the PS phase as compared to SEBS-30PS. This is possibly because of slightly greater ease of the segmental mobility of PS chains in SEBS-50PS, due to co-continuity.

The DSC results are not very conclusive. The *T*_g_ values of the PS phase of all the blends in the DSC results are very similar, unlike the ones obtained from DMA studies, possibly because as compared to DSC, DMA provides more useful information on molecular chain structure and interfacial interactions in multi-phase polymeric materials [49]. Using the same line of reasoning, not much significance can be given to the 2 °C difference in *T*_g_ of the PS phase of SEBS-30PS and SEBS-50PS in the DSC results.

Figure 7b shows the TGA plots of SEBS, PS and the blends. All compositions degrade above 400 °C. SEBS has a higher thermal stability than PS, as is evident from the higher values of *T*_onset_, *T*_25_ and *T*_50_ (Table 3). With the addition of an increasing amount of PS, thermal stability decreases because SEBS-10PS, SEBS-30PS and SEBS-50PS have progressively lower values of *T*_onset_, *T*_25_ and *T*_50_. However, the lowering of thermal stability with the increase in PS content would have no adverse effect during the processing of these materials, because of the high values of *T*_onset_. 

### 3.4. Dynamic Rheology

**Figure 8:**
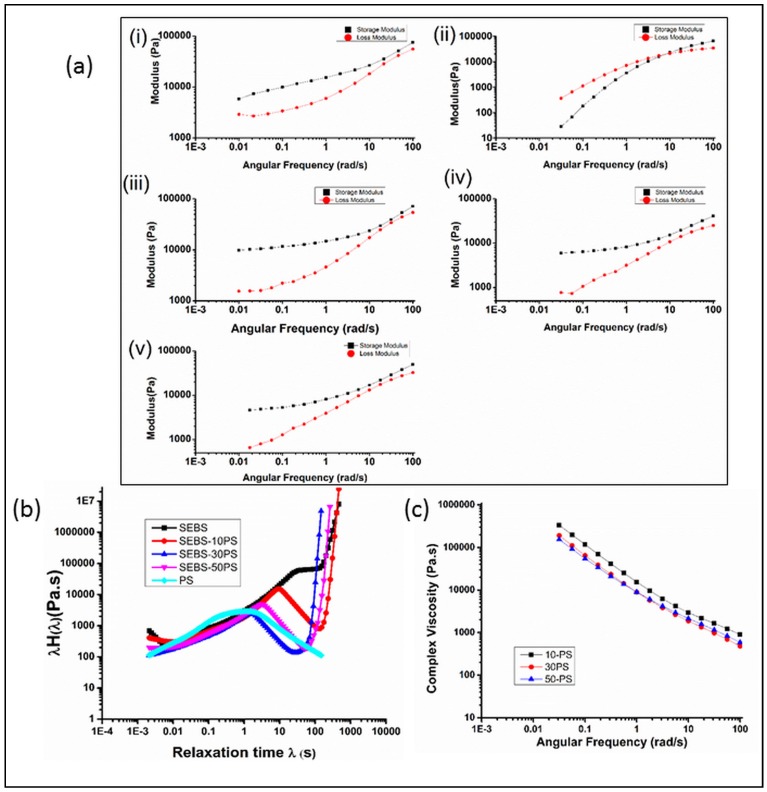
(**a**) Storage modulus and loss modulus plots of (i) SEBS, (ii) PS, (iii) SEBS-10PS, (iv) SEBS-30PS and (v) SEBS-50PS; (**b**) weighted relaxation spectrum; (**c**) complex viscosity curves.

Figure 8a shows the storage modulus (*G*’) and loss modulus (*G*”) plots of SEBS, PS and the blends. PS shows a cross-over frequency of about 7 rad/s. SEBS and the blends, however, show a predominance of *G*’ in the entire frequency range used for he studies. Frequency dependence of the storage modulus decreases in the low frequency region, with all blends showing non-terminal behavior. Such features are attributed either to a slow relaxation process, typical of elastomeric materials [50], or to the presence of a yield stress, which prevents the relaxation [51] of the elastomeric phase. However, research groups [28,52,53] working on rheological studies on SBS and SEBS have proposed that if the deformation rate is sufficiently low, relaxation of the elastomeric phase does occur even at small shear strains. 

In the frequency region close to the cross-over frequency of PS, the *G*’ and *G*” curves of each of the other compositions approach one another. This indicates a second mode of relaxation, corresponding to the PS phase of the copolymer and the blends. 

The two modes of relaxation are more obvious in the weighted relaxation spectra λH(λ) (Figure 8b) obtained from frequency sweep data using the nonlinear Tikhonov regularization method [13,54,55]. The weighted relaxation spectrum of PS shows a peak at about 1 s. In SEBS, there is no distinct peak corresponding to PS relaxation, but a very small hump appears at around 31 s. This is because there is no phase separated PS, and the entire PS phase is a part (only 18% by weight) of the copolymer chains, in the form of domains serving as physical cross-links connecting the elastomeric network. Such physically cross-linked domains have hindered mobility and slow relaxation as compared to chains of neat PS. In the blends, there is a single peak corresponding to PS relaxation. The absence of two peaks corresponding to the PS domains and the phase separated PS aggregates may be attributed to the combined effects of partial miscibility of the added PS and strong interaction of the phase-separated PS with SEBS, as also suggested by the *T*_g_ values of the PS blends. Partial miscibility of the added PS results in an increase in domain size, which adversely affects the properties arising from the inherent morphology of SEBS. Thus, the larger sized PS domains have less hindered mobility and a faster relaxation than the domains of SEBS. The phase separated fibrillar PS, on the other hand, has a slower relaxation than neat PS, due to its interaction with SEBS. The combined result of these two effects is a single peak observed at a higher value of relaxation time than neat PS, but lower than that of the PS phase of neat SEBS. 

The sharp rise in the curves in the region beyond 100 s indicates another mode of relaxation corresponding to the EB phase. The peak corresponding to this relaxation is not observed, either because the time duration of the experiment was not long enough for completion of the relaxation process of the elastomeric phase, or because of the presence of a yield stress. However, based on earlier studies [28,52,53], the former possibility is the probable cause. The presence of such multiple modes of relaxation in complex structured materials, including ones in which one of the relaxation modes is prevented/retarded, has also been reported by other research groups [13,14].

The slow relaxation of the elastomeric phase of these materials would result in time-dependent shrinkage in secondary processing operations. This could be one of the reasons why in our earlier studies on foaming of SEBS and SEBS/PS blends [9,38], selective foaming of the elastomeric phase had resulted in foams exhibiting prominent shrinkage with time.

### 3.5. Shear Rheology

**Figure 9:**
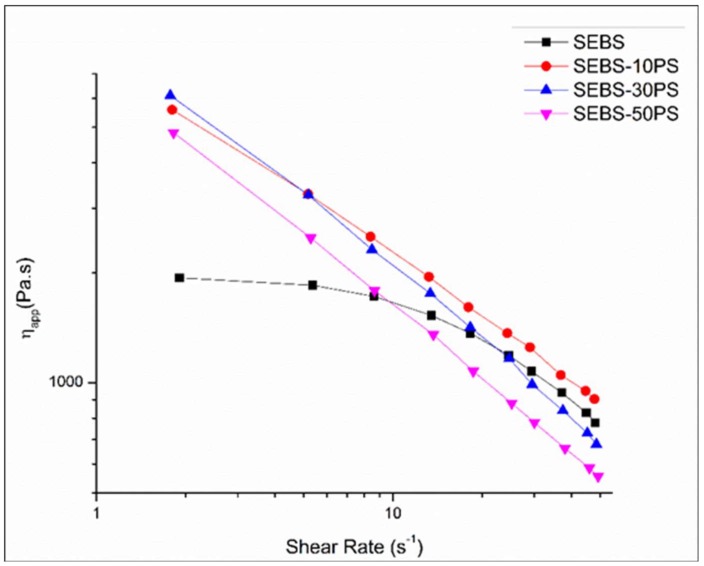
MiniLab relative rheological measurements for SEBS and blends.

Figure 9 shows the variation of η_app_ with $\dot{\gamma }$_app_ for SEBS and the blends. Unlike SEBS, which shows a Newtonian plateau at lower shear rates, the blends show shear thinning behavior in the entire range of shear rates used for studies. At times, experimental error due to slip in the rheometer leads to such an absence of the Newtonian plateau at low shear rates. However, in the present studies, a similar absence of the Newtonian plateau was also observed in the complex viscosity curves (Figure 8c). Furthermore, in dynamic rheology experiments at 200 °C, the sample was found to be tacky, adhering well to the plates, which is crucial for the no slip condition. The absence of the Newtonian plateau in both dynamic and shear rheology plots of the blends, the tacky nature of the samples during dynamic rheology experiments and the absence of breaks in the rheological curves indicate the no slip condition [56,57]. Hence, the possibility of experimental error has been ruled out.

The rheological curves of the blends and the shear thinning region of the rheological curves of SEBS and PS were fitted with the Oswald-de Waele power-law (Equation (13)).

(13) $$\eta_{\mathrm{app}}=K{{\dot{\gamma }}_{\mathrm{app}}}^{n-1}$$

where *K* is the consistency and *n* is the power law exponent.

The values of apparent viscosity at 50 s^−1^ (which falls within the shear rate range used in conventional extrusion processing) [58,59] obtained from the curves were compared with the values predicted by the logarithmic additive rule (Equation (14)) used for miscible blends [60].

(14) $$\eta_{l}=w_{\mathrm{SEBS}}\log\eta_{\mathrm{SEBS}}+w_{\mathrm{PS}}\log\eta_{\mathrm{PS}}$$

where η_SEBS_ and η_PS_ are the apparent viscosities of SEBS and PS at 50 s^−1^ obtained from the curves, η_l_ is the viscosity value at 50 s^−1^ given by the logarithmic additive rule of mixtures and *w*_SEBS_, *w*_ps_ are the weight fractions of SEBS and PS respectively.

All blends showed significant deviations from the values predicted by the logarithmic additive rule attributed to the phase separated aggregates. They were further analyzed with a simple additive rule, and the deviation Δη_50_ (obtained from Equations (15) and (16)) was used to understand the effect of the dispersed phase on the flow behavior.

(15) $$\Delta \eta_{50}=\eta_{b}-\eta_{m}$$

(16) $$\eta_{m}=\eta_{\mathrm{SEBS}}w_{\mathrm{SEBS}}+\eta_{\mathrm{PS}}w_{\mathrm{PS}}$$

where η_b_ is the apparent viscosity of the blend at 50 s^−1^ obtained from the curve and η_m_ is the viscosity value at 50 s^−1^ given by the simple additive rule of mixtures (Equation (16)). Use of the deviation of shear viscosity from the prediction of the simple additive rule of mixtures to determine the effect of the dispersed phase on the flow behavior of immiscible blends has been reported by other research groups [18,21].

The values of *K*, *n*, η_50_ of the different compositions are given in Table 4.

SEBS-10PS shows a positive deviation of viscosity from the additive rule of mixtures. This may be attributed to the predominance of the increase in the domain size. The ease of flow is a characteristic property of thermoplastic elastomers. The increase in domain size affects the inherent ease of flow of SEBS, thus increasing the viscosity. SEBS-30PS and SEBS-50PS show a negative deviation of viscosity from the additive rule of mixtures. This may be attributed to the predominance of phase separated PS aggregates. The low viscosity phase separated molten PS aggregates facilitate slippage of the SEBS chains, thereby promoting flow, which is manifested as lowering of viscosity. In the case of SEBS-30PS and SEBS-50PS, the phase separated PS is in the form of aligned fibrils, which provide a larger area of contact and are more effective in promoting flow than the small number of randomly-distributed irregularly-shaped PS aggregates in SEBS-10PS. Thus, in SEBS-30PS and SEBS-50PS, the effect of phase separated PS is far more prominent than in SEBS-10PS.

## 4. Energy Requirements for Processing

The steady state torque values (*T*_s_) during the production of the different compositions are given in Table 4. An increase in PS content from 10%–50% resulted in a lowering of *T*_s_ by around 30%, indicating lower energy consumption during mixing. Furthermore, lowering of η_50_ with the increase in PS content (Table 4) implies that blends with higher PS content have a greater ease of flow and a lower energy requirement in mixing. Thus, an increase in PS content results in lowering of the energy requirement for compounding. This increases the cost effectiveness and sustainability of these materials.

## 5. Conclusions

- When SEBS is blended with PS, a portion of the added PS increases the domain size of the copolymer, while the remaining portion phase separates. Blends of higher PS content show a fibrillar microstructure.
- An increase in PS content results in a remarkable increase in tensile modulus and an unexpected rise in *T*_g_ of the PS phase, attributed to the fibrillar microstructure in which the fibrils have a strong interaction with SEBS.
- All blends show two modes of relaxation corresponding to the PS phase and the elastomeric phase. The long relaxation time of the elastomeric phase indicates the tendency of these blends to undergo time-dependent shrinkage in secondary processing operations.
- An increase in PS content of the blends results in lower values of viscosity and energy requirement for mixing, indicating easier flow and more sustainable processing. The blends with a fibrillar microstructure have lower than expected values of viscosity attributed to additional slippage provided by the aligned low viscosity fibrils to the elastomeric chains.

## Figures and Tables

**Table 1:** Tensile properties of styrene-ethylene-butylene-styrene (SEBS) and the blends.

Composition	wt % of SEBS	wt % of PS	*E*_20_ (MPa)	UTS (MPa)	EAB (%)	Toughness (MJ/m^3^)
SEBS	100	0	2.52 ± 0.35	11.18 ± 1.1	3435.24 ± 300.69	282.70 ± 3.82
SEBS-10PS	90	10	2.38 ± 0.32	11.85 ± 0.71	2185.23 ± 188.94	170.40 ± 2.56
SEBS-30PS	70	30	9.80 ± 0.62	15.64 ± 1.97	685.4 ± 61.91	97.22 ± 6.54
SEBS-50PS	50	50	27.71 ± 1.52	21.62 ± 0.56	182.46 ± 22.99	4.74 ± 0.88

**Table 2:** Comparison of *T*_g_ values obtained from DSC plots and loss modulus plots.

Composition	Glass transition temperature(s) (°C)			
	*T*_g_ of EB Phase		*T*_g_ of PS Phase	
	DSC Studies	Loss modulus peak	DSC Studies	Loss modulus peak
SEBS	−44	−29	102	93
PS	NA	NA	99	106
SEBS-10PS	−45	−29	102	97
SEBS-30PS	−45	−28	102	116
SEBS-50PS	−42	−26	104	112

**Table 3:** Degradation temperatures of SEBS and the blends.

Composition	*T*_onset_ (°C)	*T*_25_ (°C)	*T*_50_ (°C)
SEBS	429	439	451
PS	410	415	427
SEBS-10PS	423	431	443
SEBS-30PS	417	427	440
SEBS-50PS	413	423	435

**Table 4:** Rheological parameters, shear viscosity and torque values.

Composition	*K* (Pa·s^n^)	*n*	η_50_ (Pa·s)	Δη_50_ (Pa·s)	*T*_s_ (Nm)
SEBS	6347	0.47	777.11	NA	0.54
SEBS-10PS	8159	0.43	902.74	135.13	0.42
SEBS-30PS	9648	0.33	679.10	−69.50	0.36
SEBS-50PS	7527	0.34	554.76	−174.83	0.29
PS	13,134	0.24	682.07	NA	0.52
